# Acute Cardiac Disorder or Pneumonia and Concomitant Presence of Pulmonary Embolism

**DOI:** 10.1371/journal.pone.0047418

**Published:** 2012-10-16

**Authors:** Martin Rohacek, Zsolt Szucs-Farkas, Carmen A. Pfortmüller, Heinz Zimmermann, Aristomenis Exadaktylos

**Affiliations:** 1 Department of Emergency Medicine, Inselspital, University Hospital Bern, Bern, Switzerland; 2 Department of Diagnostic, Interventional and Pediatric Radiology, Inselspital, University Hospital Bern, Bern, Switzerland; 3 Department of Internal Medicine, Inselspital, University Hospital Bern, Bern, Switzerland; Heart Center Munich, Germany

## Abstract

**Purpose:**

To determine the frequency of apparent acute pulmonary embolism (PE) and of concomitant disease in computed tomography pulmonary angiography (CTPA); to compare the frequency of PE in patients with pneumonia or acute cardiac disorder (acute coronary syndrome, tachyarrhythmia, acute left ventricular heart failure or cardiogenic shock), with the frequency of PE in patients with none of these alternative chest pathologies (comparison group).

**Methods:**

Retrospective analysis of all patients who received a CTPA at the emergency department (ED) within a period of four years and 5 months.

**Results:**

Of 1275 patients with CTPA, 28 (2.2%) had PE and concomitant radiologic evidence of another chest disease; 3 more (0.2%) had PE and an acute cardiac disorder without radiological evidence of heart failure. PE was found in 11 of 113 patients (10%) with pneumonia, in 5 of 154 patients (3.3%) with an acute cardiac disorder and in 186 of 1008 patients (18%) in the comparison group. After adjustment for risk factors for thromboembolism and for other relevant patient’s characteristics, the proportion of CTPAs with evidence of PE in patients with an acute cardiac disorder or pneumonia was significantly lower than in the comparison group (OR 0.13, 95% CI 0.05–0.33, p<0.001 for patients with an acute cardiac disorder, and OR 0.45, 95% CI 0.23–0.89, p = 0.021 for patients with pneumonia).

**Conclusion:**

The frequency of PE and a concomitant disease that can mimic PE was low. The presence of an acute cardiac disorder or pneumonia was associated with decreased odds of PE.

## Introduction

Acute pulmonary embolism (PE) is a disease that can frequently cause dyspnea, chest pain, fainting, and hemoptysis [1], and can be mimicked by other pulmonary and cardiac diseases. Its annual incidence is approximately 3 to 6 cases per 10,000 persons in the general population, and the case fatality rate ranges from less than 1% to 60% [2], [3], [4]. Validated clinical decision rules help to estimate pretest probability, and PE can be safely excluded in patients with low to intermediate probability for PE, but without elevated D-dimers [5], [6], [7]. Although there is evidence of a raised risk for PE in hospitalized patients with heart failure and in patients with a recent respiratory infection [8], [9], little is known about the presence of concomitant PE in patients presenting at the emergency department (ED) with an acute cardiac disorder or with pneumonia. This information could help physicians to estimate the pretest probability of PE in patients in whom a disease that may cause the patient’s symptoms has already been found, but where concomitant PE is still suspected. In these situations, clinical arguments gain additional importance, because D-dimers are often elevated due to pneumonia or heart failure, and may not be useful in excluding the presence of concomitant PE [10]. The aim of this study was to determine the frequency of patients with PE diagnosed by computed tomography pulmonary angiography (CTPA) who also had radiological evidence of another chest disease than PE, to determine the frequency of patients with concomitant presence of PE and pneumonia or an acute cardiac disorder, and to compare the proportion of CTPAs with evidence of PE in patients suffering from pneumonia or an acute cardiac disorder with patients who received a CTPA, but who did not suffer from these diseases.

## Methods

### Patients

For our retrospective analysis, we included all patients aged ≥16 years, who received CTPA to exclude or confirm PE at the ED of a tertiary care hospital, caring for about 30,000 patients per year, from January 2008 through to May 2012. At our ED, every decision to perform a CTPA has to be discussed with a senior physician. A senior physician is always present at the ED, even at night and during the weekend. CTPAs can be performed 24 hours a day 7 days a week, and a radiologist is always present. All CTPAs were included, regardless of other additional reasons for the same computed tomogram (CT) (e.g. consequences of trauma, suspected pulmonary diseases, cancer, or aortic dissection). For all patients who received a CTPA in the study period, the patient’s characteristics and the diagnosis at discharge from the emergency department were identified in our internal database. To estimate the patient’s pretest probabilities for PE before CTPA, the simplified revised Geneva score [6] (see Table S1) was calculated retrospectively.

All patients were examined with a 16-row CT system (Somatom Sensation 16, Siemens, Forchheim, Germany). All CTPAs were reviewed by at least two radiologists, one of them a board certified radiologist with a minimum of 5 years of experience with CTPA. To measure D-dimers, the enzyme-linked fluorescent immunoassay VIDAS^©^ (bioMérieux, Marcy l’Etoile, France) was used, with a cutoff value of 500 ng/mL. PE was defined as evidence of filling defects anywhere in the pulmonary arteries, from the pulmonary trunk down to the first subsegmental level in at least two 1.5 mm thick transverse images in CTPA. CT evidence of chest disease other than PE in CTPA was defined as follows: pulmonary or mediastinal nodules or mass (by definition, larger than 1 cm), consolidation or atelectasis with evidence of enhancing pulmonary arteries (i.e., not attributable to PE or pulmonary infarction), pleural disease including significant effusion >1 cm that was not attributable to PE, signs of left ventricular heart failure (interstitial or alveolar pulmonary edema, pleural effusion and dilated left ventricle), diseases of the aorta, and evidence of significant skeletal disease (i.e. fracture, infection, or metastatic disease). Patients were allocated to the pneumonia group, to the acute cardiac disorder group, and to the comparison group. Pneumonia was defined as evidence of clinical signs and symptoms of pneumonia and evidence of infiltrate in CT that was not attributable to pulmonary infarction due to PE, and a final diagnosis of pneumonia was made before leaving the ED. An acute cardiac disorder was defined as the diagnosis before leaving the ED of acute coronary syndrome (ACS), acute left ventricular failure or cardiogenic shock, or tachyarrhythmia. ACS was defined as the presence of clinical and electrocardiographic signs of myocardial ischemia, and evidence of a culprit lesion in coronary angiography. Left ventricular failure was defined as acute or worsening clinical signs and symptoms of heart insufficiency and evidence of pulmonary edema, pleural effusion or enlargement of the left atrium or ventricle in CT. Cardiogenic shock was defined as clinical signs of cardiogenic shock and echocardiographic signs of left ventricular heart failure. Tachyarrhythmia was defined as electrocardiographic evidence of supraventricular tachycardia, tachycardic atrial fibrillation, tachycardic atrial flutter, ventricular tachycardia, or ventricular fibrillation. All patients who failed to meet the criteria of pneumonia or acute cardiac disorder were allocated to the comparison group.

### Statistical Analysis

Statistical analysis was performed by an independent statistician. Statistical package R (The R Foundation for Statistical Computing Version 2.12.2) was used. Descriptive statistics between study groups comprised counts and percentages for categorical variables and median with interquartile range (IQR) for the non-normally distributed age values. P values for categorical variables were calculated by Chi-square tests. P values for age were determined by using the Kruskal-Wallis test. Adjusting for multiple comparisons was performed. Logistic regression was used to compare the proportion of CTPAs with evidence of PE of patients with pneumonia and with an acute cardiac disorder to the proportion of CTPAs with evidence of PE in CTPAs of the comparison group. A difference with a p value of <0.05 was considered to be significant. Significance was adjusted for risk factors for thromboembolism (i.e. age, sex, surgery during the previous month, hospitalization during the previous month, active cancer, history of thromboembolism, tachycardia, signs of deep venous thrombosis, hemoptysis, intake of oral contraceptives [11], and current autoimmune disorder [12]), for low or intermediate pretest probability but not measured D-dimers, and for ordering CTPAs to evaluate the patient for PE only and not for additional chest pathologies.

### Ethical Statement

The study was performed according to the convention of Helsinki, and was approved by the institutional review board, that waived the need for the patient’s informed consent.

## Results

During four years and five months, 1275 CTPAs were performed in patients from the ED. One hundred sixteen (9%) patients were referred by a primary or secondary hospital, and of these, 68 (5%) patients have been hospitalized in the other hospital prior to referral to our ED. The patients’ characteristics are shown in Table 1. One thousand two hundred and forty-nine (98%) cases had low or intermediate pretest probability for PE, and in 504 (40%) cases with low or intermediate pretest probability, D-dimers were not elevated (n = 69), or not measured (n = 435). Thus, 771 (60%) cases had a definite indication for CTPA.

**Table 1:** Characteristics of Patients.

	Pneumonia group	P value*	Acute cardiacdisorder group	P value†	Comparison group
	n = 113		n = 154		n = 1008
Age, median (IQR)	62 (45–73)	0.97	70 (59–77)	<0.001	63 (49–73)
Age >65 years, n (%)	53 (47)	0.45	93 (60)	<0.001	430 (43)
Male sex, n (%)	63 (56)	0.69	94 (61)	0.3	537 (53)
Sign of deep vein thrombosis‡, n (%)	3 (3)	0.1	7 (5)	0.14	90 (9)
Cancer, n (%)	27 (24)	0.043	14 (9)	0.043	158 (16)
Tachycardia§, n (%)	48 (42)	<0.001	76 (49)	<0.001	247 (25)
Hemoptysis, n (%)	8 (7)	0.04	2 (1)	0.42	29 (3)
History of thromboembolism, n (%)	13 (12)	1	13 (8)	0.8	114 (11)
Surgery||, n (%)	3 (3)	0.64	6 (4)	0.64	59 (6)
Hospitalization¶, n (%)	11 (10)	0.89	11 (7)	0.96	77 (8)
Autoimmune disorder, n (%)	11 (10)	0.041	3 (2)	0.21	45 (4)
Oral contraceptives, n (%)	2 (2)	1	2 (1)	1	23 (2)
Cardiac arrest/CPR, n (%)	0 (0)	0.11	20 (13)	<0.001	29 (3)
Evaluating for PE only**, n (%)	48 (43)	0.01	92 (60)	0.65	579 (57)
Referred by another hospital	13 (12)	0.58	17 (11)	0.58	86 (9)
Pretest probability††					
Low (0–1 points), n (%)	36 (32)	0.032	44 (29)	0.002	440 (44)
Intermediate (2–4 points), n (%)	74 (66)	0.049	106 (69)	0.003	549 (55)
High (>4 points), n (%)	3 (3)	0.8	4 (3)	0.8	19 (2)
Low/Intermediate, D-dimers not					
elevated‡‡, n (%)	4 (4)	0.58	4 (3)	0.36	61 (6)
Low/Intermediate, D-dimers not					
measured, n (%)	55 (49)	0.001	59 (38)	0.13	321 (32)

Pneumonia was defined as evidence of clinical signs and symptoms of pneumonia and evidence of infiltrate in CTPA that was not attributable to pulmonary infarction due to PE, and a final diagnosis of pneumonia before leaving the ED. An acute cardiac disorder was defined as the diagnosis of acute coronary syndrome, confirmed by coronary angiography (ACS), acute left ventricular failure or cardiogenic shock, or tachyarrhythmia before leaving the ED. All patients who failed to meet the criteria of pneumonia or acute cardiac disorder were allocated to the comparison group. IQR denotes interquartile range. CPR denotes cardiopulmonary resuscitation.

* Pneumonia group vs. comparison group;

† acute cardiac disorder group vs. comparison group;

‡ pain or swelling of a limb;

§ ≥100 beats/minute;

|| history of surgery during previous month;

¶ history of hospitalization during previous month;

** CTPAs that was ordered to evaluate the patient for PE only and not for an additional disease such as consequences of trauma, suspected pulmonary disease, cancer or aortic dissection;

†† estimated by using simplified revised Geneva score (see Table S1);

‡‡ <500 ng/mL.

**Figure 1:**
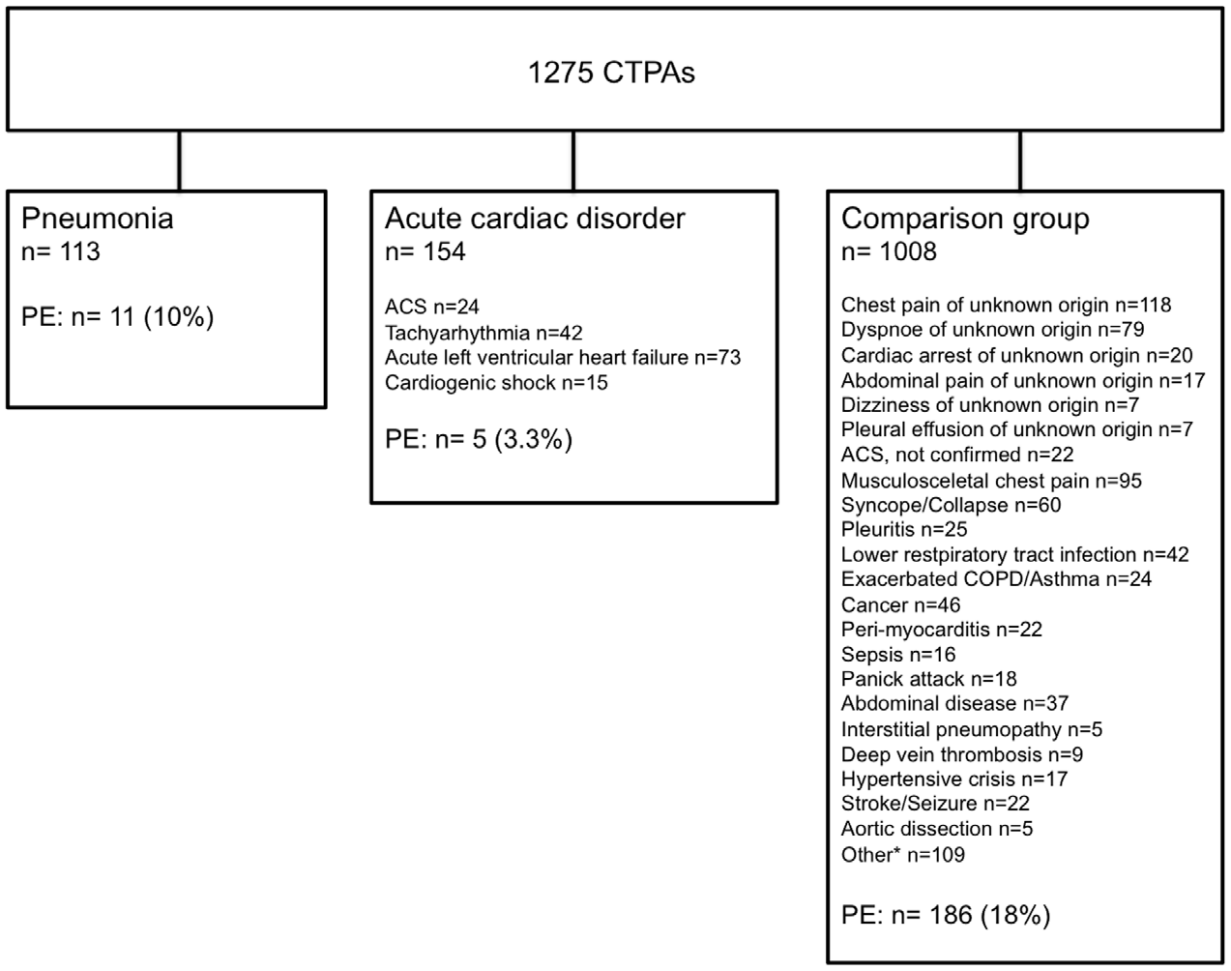
Frequencies of PE in patients with pneumonia, acute cardiac disorder, and in patients of the comparison group. CTPA denotes computed tomography pulmonary angiography. PE denotes acute pulmonary embolism. ACS, not confirmed denotes suspected acute coronary syndrome that was not confirmed by coronary angiography. Patients in the comparison group had either PE or one of the listed diagnoses at discharge from the emergency department. *Other: empyema, hematoma, leg pain or leg swelling of unknown origin, nephrotic syndrome, pneumothorax, obesity hypoventilation syndrome, sickle cell crisis, sinusitis, tuberculosis, malaise of unknown origin, fever of unknown origin, paralysis, elevated D-dimers of unknown origin, hemoptysis of unknown origin, cough of unknown origin, epiglottitis, hepatic encephalopathy, coma of unknown origin, anemia, ketoacidosis, anaphylactic reaction, struma, intoxication, spondylodiscitis, sternum infection, knee arthritis.

Two hundred and two (16%) CTPAs contained evidence of PE. Seven (3%) patients were treated by surgical removal of the embolus, and 14 (7%) received lytic therapies. In 28 (2.2%) CTPAs, there was concomitant presence of PE and radiological evidence of another chest disease, and in 3 more cases (0.2%), there was concomitant presence of PE and an acute cardiac disorder without radiological evidence of heart failure (Table 2). Of 113 patients who suffered from pneumonia, 11 (10%) had concomitant PE. Of 154 patients who suffered from an acute cardiac disorder, only five (3.3%) had concomitant PE. Of the 1008 remaining patients of the comparison group who received a CTPA, 186 (18%) had PE (Figure 1). Table 3 outlines the frequencies of PE for pretest probabilities (i.e. low, intermediate, high) in the different groups. After adjustment for risk factors for thromboembolism, for low or intermediate pretest probability but not measured D-dimers, and for ordering CTPAs to evaluate the patient for PE only and not for additional chest pathologies, the proportions of CTPAs with evidence of PE in patients with an acute cardiac disorder or pneumonia was significantly lower than in the comparison group (OR 0.13, 95% CI 0.05–0.33, p<0.001 for patients with an acute cardiac disorder, and OR 0.45, 95% CI 0.23–0.89, p = 0.021 for patients with pneumonia.

**Table 2:** Cases of concomitant presence of PE and another disease that could mimic PE.

	n =
**Evidence of chest disease in CTPA**	
CAP*	10
*P.jirovecii* pneumonia	1
Metastatic cancer	3
Relapsing AML with lymphadenopathy	1
Emphysema	5
Granulomatous pneumopathy	1
Chronic aspergilloma	1
Pulmonary laceration/rib fracture after trauma	1
Serothorax after pneumonectomy	1
Pulmonary hypertension due to HIV infection	1
Acute heart failure	1
Cardiogenic shock due to ACS	1
Spondylodiscitis	1
**Cardiac disorder without evidence in CTPA**	
STEMI	2
New onset of tachycardic atrial fibrillation	1

PE denotes pulmonary embolism. CTPA denotes computed tomography pulmonary angiography. CAP denotes community acquired pneumonia. AML denotes acute myeloid leukemia. ACS denotes acute coronary syndrome. STEMI denotes ST-elevation myocardial infarction.

* includes one case of *Mycoplasma pneumonia* infection with hemolytic anemia.

## Discussion

In our study, only 2.2% of 1275 patients admitted to the ED, who received a CTPA, had radiological evidence of a concomitant disease in addition to PE, and 2.4% of all patients had evidence of a concomitant disease in CTPA or an acute cardiac disorder in addition to PE. The presence of a disease that could mimic the symptoms of PE, such as an acute cardiac disorder or pneumonia, was associated with decreased odds of PE. In contrast to the comparison group, the frequency of PE in patients with acute cardiac disorder or pneumonia was low throughout all pretest probabilities (Table 3).

**Table 3:** Frequencies of PE.

Groups	Pretest probability for PE		
	Low	Intermediate	High
All (n = 1275)	52/520 (10%)	143/729 (20%)	7/26 (27%)
Pneumonia (n = 113)	3/36 (8%)	8/74 (11%)	0/3 (0%)
Acute cardiac disorder (n = 154)	1/44 (2%)	4/106 (4%)	0/4 (0%)
Comparison group(n = 1008)	48/440 (11%)	131/549 (24%)	7/19 (37%)

PE denotes acute pulmonary embolism. To estimate pretest probabilities, the simplified revised Geneva score was used (low probability: <2 points; intermediate probability: 2 to 4 points; high probability: >5 points, see Table S1).

It has been shown, that acute infection and recent respiratory infection, and raised inflammatory markers, are associated with increased odds of thromboembolic disease [9], [13], [14]. In one study, pneumonia was associated with PE (adjusted OR = 2.38, 95% CI 1-13-4.99) [9]. However, in that study, patients were analyzed five to 52 weeks after pneumonia, and not during acute infection. In another study, there was evidence of a relative risk of 2.15 (95% CI 2.15–2.16) for PE for hospitalized patients with heart failure, compared with patients without heart failure [8]. However, the prevalence of thromboembolism in patients with an acute heart disorder presenting to the ED is unknown. On the other hand, in the analysis of the outpatient and inpatient population with suspected PE, from which the Wells-score was derived, the possibility of a diagnosis other than PE was associated with decreased odds of PE [5], which is in line with our findings. In our study, 95% of the patients have been admitted from home. In these patients, our findings suggest that the obvious presence of a disease that could mimic the symptoms of PE makes a concomitant presence of PE unlikely. This could help to decide when to order a CTPA, particularly in patients with low or intermediate probability, as estimated by validated scores, and with elevated D-dimers.

Our study has several limitations. First, it was a retrospective analysis of a selected group of patients who received a CTPA ordered by physicians of the ED to confirm or rule out PE. However, this reflects a “real world” selection of patients presenting at our ED. Second, this investigation was a single centre study. In 40% of cases with low or intermediate pretest probability for PE, the D-dimers were not elevated or this test was not performed, even though, in 2010, all physicians received training in diagnosing PE using the Wells score and the simplified revised Geneva score in combination with the D-dimer test and CTPA. This is comparable to a multicenter study, where in one third of patients with Wells score <6 points - indicating low or intermediate pretest probability for PE-, the D-dimer test was negative or not performed, and CTPA was potentially avoidable [15]. Thus, the behavior of physicians of our ED in using guidelines might be comparable to physicians in other EDs. We note that in a survey performed in 2010 to 2011 at our ED, physicians declared that about one half of CTPAs were not only ordered out of adherence to evidence based decision rules, but also out of “fear of missing PE” [16]. It is not known if physicians of other EDs have similar motivations for ordering radiologic tests, and, therefore, the possibility of generalizing this present study might be limited.

Third, an acute cardiac disorder is more strongly associated with decreased odds of PE than is the presence of pneumonia. We cannot determine whether this was due to the selection bias in patients to receive a CTPA or due to a true elevated risk for PE in patients with pneumonia compared to patients with an acute cardiac disorder.

Fourth, to estimate the pretest probability retrospectively, we used the revised simplified Geneva score, which is normally used at our ED, although the prevalence of PE in our patients was lower than in the population from which the revised simplified Geneva score was derived (16% vs. 23%). Thus, the estimation of pretest probability might not have been exact. Therefore, we adjusted the significance of the results for the different risk factors for thromboembolism, and not for pretest probability. We could not use the Wells score, because it is not possible to determine the variable “an alternative diagnosis is less likely than PE” retrospectively.

Finally, we did not perform a follow up. Thus, we are not able to determine the number of patients with a negative CTPA who developed PE after discharge from the ED. However, a metaanalysis with patients with negative CTPA who did not receive anticoagulation showed a 3-month rate of subsequent thromboembolism of 1.4%, and a 3-month rate of fatal PE of 0.51% [17], which is similar to the results after a normal invasive pulmonary angiogram [18]. All studies of this metaanalysis used early-generation CT technology. Thus, an even lower rate of subsequent thromboembolism might be expected after CTPAs performed with a 16-row CT system that is available at our institution.

In conclusion, the frequency of the concomitant presence of PE and another disease that can mimic the symptoms of PE was low in patients who were selected to receive a CTPA at our ED. The presence of pneumonia or, most notably, an acute cardiac disorder, was associated with decreased odds of PE. Our results could further encourage physicians to avoid ordering unnecessary CTPAs.

## Supporting Information

Table S1 **Simplified Revised Geneva Score.** Simplified Revised Geneva Score: 0–1 points indicates low (8%), 2–4 points intermediate (29%), and ≥5 points high (64%) pretest probability for PE. DVT denotes deep vein thrombosis, PE denotes pulmonary embolism.
(DOCX)
