# Supplementary material for: Acute Cardiac Disorder or Pneumonia and Concomitant Presence of Pulmonary Embolism
Source: PLoS One. 2012 Oct 16;7(10):e47418. doi: 10.1371/journal.pone.0047418 (PMC3473021; doi:10.1371/journal.pone.0047418)
Supplement: Table S1 — Simplified Revised Geneva Score. Simplified Revised Geneva Score: 0–1 points indicates low (8%), 2–4 points intermediate (29%), and ≥5 points high (64%) pretest probability for PE. DVT denotes deep vein thrombosis, PE denotes pulmonary embolism. (DOCX) [file pone.0047418.s001.docx]

**Supplementary appendix**

|  | **Points** |
| --- | --- |
| Age > 65 years | 1 |
| Previous DVT or PE | 1 |
| Surgery (under general anesthesia)  or fracture (of lower limbs) within 1 month | 1 |
| Active malignant condition (solid or hematologic, currently active or considered cured < 1 year | 1 |
| Unilateral lower-limb pain | 1 |
| Hemoptysis | 1 |
| Heart rate, beats/minute | 1 |
| 75-94 | 1 |
| ≥95 | 1 |
| Pain on lower-limb deep venous palpation  and unilateral edema | 1 |
